# Eliciting Opinions on Health Messaging During the COVID-19 Pandemic: Qualitative Survey Study

**DOI:** 10.2196/39697

**Published:** 2023-04-27

**Authors:** Sienna Ruiz, Uzoma Charles Okere, Michelle Eggers, Catina O'Leary, Mary Politi, Fei Wan, Ashley J Housten

**Affiliations:** 1 Division of Public Health Sciences Department of Surgery Washington University in St. Louis St. Louis, MO United States; 2 Health Literacy Media St. Louis, MO United States

**Keywords:** COVID-19, health messaging, rural populations, urban populations, communication, health information, messaging, dissemination, health equity, prevention, implementation

## Abstract

**Background:**

Effective public health messaging has been necessary throughout the COVID-19 pandemic, but stakeholders have struggled to communicate critical information to the public, especially in different types of locations such as urban and rural areas.

**Objective:**

This study aimed to identify opportunities to improve COVID-19 messages for community distribution in rural and urban settings and to summarize the findings to inform future messaging.

**Methods:**

We purposively sampled by region (urban or rural) and participant type (general public or health care professional) to survey participants about their opinions on 4 COVID-19 health messages. We designed open-ended survey questions and analyzed the data using pragmatic health equity implementation science approaches. Following the qualitative analysis of the survey responses, we designed refined COVID-19 messages incorporating participant feedback and redistributed them via a short survey.

**Results:**

In total, 67 participants consented and enrolled: 31 (46%) community participants from the rural Southeast Missouri *Bootheel*, 27 (40%) community participants from urban St Louis, and 9 (13%) health care professionals from St Louis. Overall, we found no qualitative differences between the responses of our urban and rural samples to the open-ended questions. Participants across groups wanted familiar COVID-19 protocols, personal choice in COVID-19 preventive behaviors, and clear source information. Health care professionals contextualized their suggestions within the specific needs of their patients. All groups suggested practices consistent with health-literate communications. We reached 83% (54/65) of the participants for message redistribution, and most had overwhelmingly positive responses to the refined messages.

**Conclusions:**

We suggest convenient methods for community involvement in the creation of health messages by using a brief web-based survey. We identified areas of improvement for future health messaging, such as reaffirming the preventive practices advertised early in a crisis, framing messages such that they allow for personal choice of preventive behavior, highlighting well-known source information, using plain language, and crafting messages that are applicable to the readers’ circumstances.

## Introduction

### Background

Since the emergence of SARS-CoV-2 in 2019 and its resultant disease, COVID-19, public health communication has rapidly adapted to constantly changing information. Adding complexity to public health messaging, the arrival of variant strains, vaccinations [1], and regional differences in the timing and intensity of disease spread [2] have shifted the course of the pandemic. Given the rapid developments in public health practices, scientific innovations, and epidemiological trends, effective health messaging remains critical for improving public awareness and informing health protocols [3].

In 2002, the US Centers for Disease Control and Prevention (CDC) published manuals such as the Crises and Emergency Risk Communication [4]. The Crises and Emergency Risk Communication manual advocates for trusted sources to be first, be right, be credible, support action, and show respect and empathy toward its audience [4]. However, these principles were not fully applied in the United States in response to the onset of the COVID-19 pandemic. Government and health officials were often not the first to speak on COVID-19, leading the public to question information sources. Limited efforts were made to adapt information to evolving circumstances, and complex concepts such as the risks related to COVID-19 were difficult to convey [4].

In addition, rural populations reported distinct beliefs about the COVID-19 pandemic [5], were overall less likely to engage in COVID-19 preventive health behaviors [6,7], and responded differently from urban populations to specific dissemination strategies for health promotion [8]. Specifically, researchers have found that rural populations may be exposed to various structural barriers (eg, fewer educational opportunities [9]) and express political differences (eg, higher beliefs in individualism [10]) that contribute to them having higher levels of distrust related to preventive behaviors such as vaccination and masking [10,11] than urban populations. Such differences persist because rural communities have experienced more severe impacts of the COVID-19 pandemic than urban communities owing to increased rates of poverty, comorbidities, and low access to health resources [9]. To equitably direct health resources, including health messaging, an understanding of the underlying individual and social contexts among geographically diverse populations is required [12].

### Goals of This Study

To address missteps in health messaging early in the pandemic, researchers and public health professionals must examine the efficacy of health messages and identify best practices. Currently, there continues to be a need for efficient health messaging regarding COVID-19 risks, treatment, prevention, and vaccination [4]. Attributes such as clarity, concision, legibility, attractiveness, realistic guidance, and emotional appeal are essential components of successful COVID-19 health messaging [3,13,14]. In this study, we aimed to identify opportunities to improve COVID-19 messages for community distribution by health officials and summarize the findings to inform future messaging. Findings from this study can improve how stakeholders approach health messaging design in various contexts and inform the dissemination of future health messaging that incorporates perspectives from stakeholders across urban and rural settings.

## Methods

### Setting

Community participants were recruited from 2 regions of Missouri, Southeast Missouri (the *Bootheel*) and the St Louis metropolitan region (STL), and a small group of health professionals were recruited from St Louis. In the Bootheel, most care is provided by federally qualified health clinics in the absence of major hospitals [15]. The Bootheel has higher rates of poverty, higher chronic disease burden, and more older people as well as lower educational attainment than other regions of Missouri [16]. Outside the cities of St Louis and Kansas City, counties in the Bootheel have some of the highest number of Black populations in the state [17]. In urban STL, access to health care resources is mediated by racialized segregation, with the majority Black populations in North St Louis facing higher rates of comorbidities, increased poverty, and more limited availability of health care resources than the majority White populations in South St Louis [18] despite the presence of several major health care institutions in the area at large. Both the Bootheel and St Louis have similarly low levels of health literacy [16].

### Message Review and Identification

From July 2020 to September 2020, the research team reviewed the existing public health messages to be used in the surveys. Two research team members used a search engine (eg, Google [Google LLC]) and social media (eg, Facebook [Meta Platforms, Inc] and Instagram [Meta Platforms, Inc]) to identify local, state, national, and international COVID-19 public health messages. Following the full team review, we chose 2 messages in each of the following two types: (1) risk presentations and (2) infographics. A total of 4 messages were chosen because they varied in content, format, and imaging, and they were widely used in the media. Only 4 messages were selected to ensure adequate time in the web-based interview to fully explore how participants responded to the health information in 2 messages, along with their preferences associated with the overall content, format, and imaging in the selected messages. The selected messages were focused on prevention protocols and presented COVID-19 risk using various visual communication strategies. Their sources represented a range of experts (eg, the World Health Organization [WHO], the CDC, and Doctor of Medicine groups) and are described in detail in Table 1.

**Table 1:** Summary of the messages for each message set^a^.

	Message set 1			Message set 2	
	1A	1B	2A		2B
Title	“Stop the Spread of Germs”	“COVID-19 Risk Index”	“Avoid the Three Cs. Be Aware of Different Levels of Risk in Different Settings”		“Two Metres or One: What Is the Evidence for Physical Distancing in Covid-19?”^b^
Citation	[19]	[20]	[21]		[22]
Content and text	Depicts protocols for preventing the spread of COVID-19 and other respiratory viruses, including washing hands, wearing a face covering, and staying 6 ft away from others	Divides common activities into columns based on risk level (ranging from “low” to “high” risk) Lists 4 factors that affect risk Tells viewers to wear a mask	Describes the 3 Cs—3 factors that increase the likelihood of spread: “crowded places, close-contact settings, and confined and enclosed spaces” States what actions the viewer should take		Depicts the risk of COVID-19 transmission based on multiple factors: whether people are silent, speaking, or shouting or singing; whether face coverings are worn; how long the contact lasts; the level of occupancy; and ventilation quality
Images	Simplified drawings of people performing the recommended protocols	5 columns colored according to the risk level and filled with black icons representing different activities A black and white image of a mask	3 circles depicting simplified drawings of the 3 Cs A Venn diagram of the 3 Cs Small black drawings of the recommended protocols		Cells in tables filled with different colors depending on the risk level
Colors	Blue, green, and gold	Shades of green, orange, yellow, and red	Blue, yellow, and red		Red, yellow, and green

^a^1A, 1B, 2A, and 2B are the image abbreviations used.

^b^On the basis of the figure presented in the study “Two Metres or One: What Is the Evidence for Physical Distancing in Covid-19?” [22].

### Ethics Approval

All surveys and interview guides were approved by the Washington University Institutional Review Board (#202010069). All research procedures were approved by the Washington University School of Medicine Institutional Review Board.

### Surveys

Given the potential differences between urban and rural populations, we surveyed populations from 2 distinct regions, urban St Louis, Missouri, and the rural Southeast Missouri *Bootheel*, to assess preferences for COVID-19 messaging. We used a purposive sample recruitment approach in both the St Louis and Bootheel regions because our research team had preexisting connections with community organizations that could aid recruitment in both areas. Our study design followed the principles of pragmatic health equity implementation science by surveying members of the general public in each region and health care professionals on their message preferences to inform the immediate development of new messages [12,23]. We situated our findings on COVID-19 health messaging within the social and economic contexts that the participants reported during their survey.

We surveyed participants to elicit their opinions on COVID-19, including their preferences for preselected COVID-19 messages. The survey session lasted an average of 1 hour for each participant. Participants received a US $50 gift card for their time.

In the context of the COVID-19 pandemic, when in-person interviews were not considered appropriate or safe, the research team operationalized a web-based approach to capture participants’ opinions. To recruit participants, the research team members broadly distributed a web-based survey link via social media (eg, Facebook, Twitter [Twitter, Inc], and Craigslist [Craigslist, Inc]). This survey collected the participants’ contact information, including their email addresses, which were then kept within an Institutional Review Board–approved, password-protected database. After a participant completed the survey and was found eligible, the study team contacted them to schedule the full survey evaluating health messages. Public participants were eligible to participate if they were (1) self-reported English speakers, (2) aged 18 to 80 years, and (3) residing in either the Bootheel or St Louis. Our age cutoff for eligibility was 80 years owing to limitations in the feasibility of recruiting older adults remotely during COVID-19 surges, concerns over access to technology among this population, and potentially differing risk reduction recommendations for older adults. Participants were considered residents of the Southeast Missouri Bootheel if they lived in Dunklin, Stoddard, Mississippi, Pemiscot, or New Madrid County. Participants were considered St Louis residents if they lived in St Louis City or County. Health care professionals were eligible to participate if they (1) were self-reported English speakers, (2) resided in either the Bootheel or St Louis, and (3) self-identified as a health professional (eg, Registered Nurse, Licensed Practical Nurse, Doctor of Medicine, or Doctor of Osteopathic Medicine). Staff reached out directly via email or phone to eligible participants and scheduled a web-based appointment on a Health Insurance Portability and Accountability Act (HIPAA)–compliant Zoom (Zoom Video Communications, Inc) account for their participation in the study. Survey data were collected and managed using the REDCap (Research Electronic Data Capture; Vanderbilt University) system hosted at the Washington University [24,25].

To reduce participant burden and enhance the feasibility of survey completion, the messages were divided into 2 sets and randomly assigned to roughly equal numbers of participants by the research team before each survey. The purpose of random assignment was not to determine differences between message sets but to evaluate participants’ opinions on multiple types of health messages.

The research team conducted the surveys with participants between November 2020 and February 2021 (Multimedia Appendix 1). The survey questions followed pragmatic and health equity guidelines by evaluating the social and economic impacts of the pandemic and eliciting real-time opinions on health messaging with the goal of improving message development later in the study. Questions 15, 16, 33, and 61 on the social and economic impacts of the pandemic and questions 7 and 11 to 14 on the exposure of the participants to COVID-19 elicited potential socioeconomic and health inequities between public samples, inspired by calls to compare health indicators and individuals’ social positions (eg, race and ethnicity, socioeconomic status, and educational attainment) to examine potential health inequities [12]. Halfway through the survey, interviewers shared their screen to show participants their assigned messages. After the participants had thoroughly reviewed the messages, the interviewers asked the participants open-ended questions on their opinions of the messages. These questions are listed as questions 35 to 46 in Multimedia Appendix 1 for public participants and questions 18 to 25 in Multimedia Appendix 2 for health care professionals. These questions aimed to identify participants’ perspectives for “tailored implementation, which builds on real-world experiences to identify the participant-identified priorities to address” [12] for the dissemination of improved health messages. The interviewers took notes that closely summarized the participants’ comments. The survey process was audio recorded, and the recordings were stored on a secure university platform.

Shorter, focused surveys were conducted with health care professionals using the same methods, but the questions were designed to capture the needs of their patient populations (Multimedia Appendix 2).

To analyze the qualitative data, the research team members used inductive thematic analysis [26,27]. Data were analyzed and managed using the NVivo software (version 20; QSR International). The purpose of this study was to evaluate participants’ opinions on health messaging. Therefore, we used inductive thematic analysis to gauge *how* participants viewed each message and the salient themes they discussed in relation to their preferences for the content, design, distribution, and other aspects of the messages. Team members familiarized themselves with the qualitative data by reading through and annotating the interviewers’ notes of each participant’s responses to open-ended questions. Following a close review of the interviewers’ notes, the research team members created a codebook to guide thematic analysis. Once the codebook was finalized, each interview was independently coded by 1 of 3 coders. Then, a separate coder reviewed each coded interview, and discrepancies were discussed and reconciled by the research team to ensure greater reliability. Team members systematically read through, annotated, and summarized each code to create the thematic findings described in the *Results* section. To qualitatively compare the themes between each participant group, we identified which themes were most salient for each group by examining the degree to which a theme recurred or was important in the sample (ie, themes were considered important if they were “new and advanced understanding, were useful in addressing real-world problems, or did both”) [28]. If the coders found similar levels of recurrence and importance of the same theme in both samples, they listed the theme as salient to both groups, and they found no qualitative difference in their analysis between the groups in relation to each theme.

We used various methods to ensure qualitative rigor [29], such as holding regular team meetings to create the codebook and checking whether coders applied codes consistently across surveys. In our meetings, we also discussed how our backgrounds (eg, from different academic disciplines), our personal experiences of the pandemic, and residing in rural or urban area shaped our approaches to coding and analysis. It was discussed in depth how most authors’ life experiences in urban areas, and 1 author’s life experiences in a rural area, influenced the research team’s understanding of the similarities and differences between urban and rural regions. We consistently examined our interpretations of the thematic results to limit any potential bias toward or against a type of region or the perpetuation of any stereotypes of urban or rural regions.

### Message Redistribution

In line with the goal of equity in the dissemination of study results to end users [12], we created new COVID-19 messages based on participants’ responses and redistributed these messages for participant feedback. Following the qualitative analysis, the research team created 3 sets of images incorporating participant feedback between March and June 2021. These messages addressed safer summer activities, postvaccination guidelines, and incentives to get vaccinated and were intended to be distributed during the summer of 2021. After the designs were finalized, we recontacted the participants asking them to complete a short survey in June 2021 gathering feedback on the new messages, including whether the new images incorporated their feedback from their initial surveys. The newly created messages and full survey on message redistribution can be found in Multimedia Appendix 3. This redistribution survey approach introduces a low-resource method for eliciting health equity implementation feedback via brief web-based surveys.

## Results

A total of 67 participants completed the study, with 31 (46%) community participants from the Bootheel, 27 (40%) community participants from the St Louis area, and 9 (13%) health care professionals from the St Louis area. Overall, 52% (35/67) of participants reviewed message set 1, and 48% (32/67) of the participants reviewed message set 2.

### Participant Characteristics

Table 2 presents the sociodemographic information of the total sample. The mean age of the Bootheel public group was younger than that of the St Louis public group (Bootheel mean age 30.3, SD 10.1 years vs St Louis metro mean age 38.0, SD 13.7 years). The health care professional group’s mean age was 34.9 (SD 7.11) years. Health care professionals were either primary care providers (eg, RNs, physicians, and medical assistants) or community health workers (eg, caregivers, social workers, and mental health program managers). Participants across both samples had similarly high levels of health literacy, incomes, and educational attainment, and most participants identified as White or Black.

In terms of COVID-19 exposure, more participants in the Bootheel knew someone close to them who tested positive for COVID-19 (19/31, 61% compared with 13/27, 48% in STL) or who was hospitalized for COVID-19 (25/31, 81% compared with 14/27, 52% in STL). More participants in the Bootheel responded that they could count on people in their neighborhood to help them (28/31, 90% compared with 16/27, 59% in STL) and go to the store for them if they were sick (25/31, 81% compared with 15/27, 56% in STL). Participants in the Bootheel rated the degree to which the pandemic created financial problems for themselves or their family higher than those in St Louis (Table 3). They were also more worried about not being able to access food or important resources, such as transportation or housing, owing to the pandemic (Table 3).

**Table 2:** Baseline participant characteristics of the final sample (N=67)^a^.

			Region				
			Public (St Louis metro area; n=27)		Public (Southeast Missouri *Bootheel*; n=31)		Health care professionals (St Louis metro area; n=9)
Age (years), mean (SD; range)			38.0 (13.7; 24-67)		30.3 (10.1; 19-68)		34.9 (7.11; 25-47)
**Gender, n (%)**							
	Man	12 (44)		20 (65)		2 (22)	
	Woman	15 (56)		10 (32)		7 (78)	
	Nonbinary	0 (0)		1 (3)		0 (0)	
**Race and ethnicity, n (%)**							
	Black	15 (56)		13 (42)		6 (67)	
	White	11 (41)		16 (52)		3 (33)	
	Hispanic or Latino	1 (4)		0 (0)		0 (0)	
	American Indian or Alaska Native	0 (0)		1 (3)		0 (0)	
	Asian or Pacific Islander	0 (0)		0 (0)		0 (0)	
	Other	0 (0)		1 (3)		0 (0)	
**Education, n (%)**							
	Less than bachelor’s degree	8 (30)		7 (23)		—^b^	
	Bachelor’s degree or higher	19 (70)		24 (77)		—	
**Yearly family income, including all sources (US $)**							
	<15,000	2 (7)		2 (6)		—	
	15,000-34,999	2 (7)		0 (0)		—	
	35,000-54,999	6 (22)		8 (26)		—	
	55,000-74,999	3 (11)		15 (48)		—	
	≥$75,000	12 (44)		6 (19)		—	
	Prefer not to say	2 (7)		0 (0)		—	
Health literacy, mean (SD; range)			14.5 (2.3; 11.0-19.0)		14.2 (2.4; 10.0-18.0)		—

^a^Totals were calculated by column.

^b^We did not collect education, income, health literacy, social, or economic data from health care professionals.

**Table 3:** Social and economic impacts of COVID-19 for the public participants.

		Region	
		Public (St Louis metro area; n=27)	Public (Southeast Missouri *Bootheel*; n=31)
**Have you ever been diagnosed with COVID-19?, n (%)**			
	Yes	8 (30)	26 (84)
	No	18 (67)	5 (16)
	Not sure or do not know	1 (4)	0 (0)
**Has anyone close to you tested positive for COVID-19?, n (%)**			
	Yes	13 (48)	19 (61)
	No	12 (44)	11 (36)
	Not sure or do not know	2 (7)	1 (3)
**How many people do you know who have had COVID-19?, n (%)**			
	None	5 (19)	0 (0)
	1	3 (11)	4 (13)
	2-5	13 (48)	18 (58)
	≥6	6 (22)	9 (29)
**Do you know anyone who has been hospitalized for COVID-19?, n (%)**			
	Yes	14 (52)	25 (81)
	No	11 (41)	5 (16)
	Not sure or do not know	2 (7)	1 (3)
**Do you know anyone who has died from COVID-19?, n (%)**			
	Yes	9 (33)	9 (29)
	No	18 (67)	20 (65)
	Not sure or do not know	0 (0)	2 (7)
**I can count on people in my neighborhood to help me if I am sick, n (%)**			
	Agree	16 (59)	28 (90)
	Disagree	11 (41)	3 (10)
**My neighbors would go to the store for me if I am sick, n (%)**			
	Agree	15 (56)	25 (81)
	Disagree	12 (44)	6 (19)
How worried have you been about not being able to afford or access food because of the COVID-19 outbreak? (on a scale ranging from 1 [not worried at all] to 5 [somewhat worried] to 10 [extremely worried]), mean (SD; range)		3.0 (2.6; 0-9)	5.6 (2.8; 0-9)
How worried have you been about access to important resources such as transportation or housing due to the COVID-19 outbreak? (on a scale ranging from 1 [not worried at all] to 5 [somewhat worried] to 10 [extremely worried]), mean (SD; range)		3.0 (3.3; 0-9)	5.3 (3; 0-9)

### Thematic Findings

#### Overview

We did not identify any qualitative differences between the participants from the St Louis region and those from the Bootheel in how they responded to the messages or in their suggestions for improving the messages. Common themes for all groups included participants’ preference to see the main COVID-19 protocols in messages, desire for personal choice with regard to COVID-19 preventive behaviors, and suggestions for clear and easily accessible source information. Although health care professionals had responses similar to those of both public samples, they more often named health literacy as a factor that could compound the patient’s perceptions and made suggestions for their specific patient populations. Qualitative results are presented in the subsequent sections with italicized interviewer notes used to summarize participants’ responses to open-ended questions in the survey.

#### Theme 1: Preference for Main COVID-19 Protocols

Many participants recognized the main COVID-19 protocols as behaviors such as hand washing, maintaining 6 ft of social distancing, and wearing a mask [30]. Most participants wanted to see or expected to see these behaviors represented in the messages. Images that were missing these messages were often viewed as incomplete by the participants. One of the participants shared the following:

Yes something is missing, they should include good ways in wearing a mask, information there that shows where a person wear a mask, not leave nose uncovered, chances of transmit[ing] the virus

Interviewer notes of the response of P54 from STL about message 2A

Another participant said the following:

What about washing hands, other preventive messages...should be part of every message

Interviewer notes of the response of P14 from STL about message 1B

#### Theme 2: Desire for Personal Choice in COVID-19 Behavioral Response

The presentation of risks across various activities appeared to resonate with participants’ interest in personal choice or the freedom to make their own choices regarding their health and safety. One of the participants said the following:

I believe people have the right to make their own choices. This isn’t telling people what to do; it just...tells them about the risk. So if you do everything they recommend, your risk is low, but it allows me to make the decision for myself.

Interviewer notes of the response of P205 from the Bootheel about message 1B

Similarly, another participant said the following:

I don’t feel like they’re telling you what to do, they’re just giving you guidance on how to avoid certain situations and getting COVID.

Interviewer notes of the response of P267 from the Bootheel about message 2A

A health care professional commented the following:

I like the spectrum rather than do this and don’t do this; more realistic [because] nothing is zero risk

Interviewer notes of the response of health care professional P156 about message 1B

Another participant said that they liked that the message “doesn’t feel too preachy” (Interviewer notes of the response of P30 from STL about message 2A).

#### Theme 3: Clear and Easily Accessible Source Information

Most participants described “good” source information as being apparently authentic because of the presence a large logo, coming from a trusted source, and including resources for follow-up. Follow-up could mean obtaining more information about the message or COVID-19 or receiving contact information on whom to call in case one experiences COVID-19 symptoms. One of the participants said the following:

[It’s missing] maybe the CDC website or something...I don’t know who this is coming from. I should trust this, I guess...it’s missing the CDC or something.

Interviewer notes of the response of P23 from STL about message 1B

Another participant said that the message should provide “a piece of contact information, such as a number to call...There should be information on who to contact if I suspect someone has COVID-19, is exhibiting symptoms” (Interviewer notes of the response of P192 from the Bootheel about message 1A). A participant also remarked that the message “had no source, web link...[I am] not likely [to follow-up]. I don’t know [the] journal and don’t see [the source] as a link” (Interviewer notes of the response of P200 from the Bootheel about message 2B).

After we asked them which sources in a provided list they used before, they then identified which source they used the most as a free-response answer. The most preferred sources among the participants in St Louis were local news; social media, such as Twitter and Facebook; the WHO; and the CDC, whereas the most preferred sources in the Bootheel were social media, the WHO, and newspapers. For health care professionals, the most preferred sources were the CDC, newspapers, and local news stations.

### Health Care Professional Findings

Health care professionals contextualized their suggestions within the applicability of the messages to their patients. They assessed whether the actions outlined in the messages were applicable to their patient populations with limited health literacy or who were older, had low income, or spoke English as a second language. One of the health care professionals said the following:

I think [telling people to stay home when they’re sick] triggers people. A lot of people...can’t do that because of their financial situation, lack of sick leave, or other things.

Interviewer notes of the response of health care professional P85 about message 1A

One of the providers gave the following answer:

A lot of it [would be confusing] for my patients, most of my patients speak Spanish.

Interviewer notes of the response of health care professionalP156 about message 1A

Another provider said the following:

For some, not everything in here might...be practical. For example, staying 6 feet apart might not be practical for people...[like for those] sharing an apartment or a house with multiple people.

Interviewer notes of the response of health care professional P264 about message 1A

A health care professional who worked in a health home answered that the advice regarding avoiding close contact would be hard because “some patients like that physical contact...Some people are also hard of hearing, so you would have to get close to them so they can hear you” (Interviewer notes of the response of health care professional P276 about message 2A).

### Findings in the Context of Health Communication Best Practices

Community participants’ suggestions for message improvement aligned with the best practices for health literacy [31]. These practices included using clear, easily understandable language; visually prioritizing the most important messages; avoiding extraneous information; sufficiently spacing out images and text; using eye-catching colors; visually representing a diverse set of people; incorporating an emotional appeal; and clearly representing the source of the message. Refer to Table 3 for participants’ quotes.

Although the health care professionals’ suggestions also aligned with the principles of health literacy, they were more likely to specifically reference the terms “literacy” or “health literacy” when gauging the potential impact of the message. For example, one of the health care professionals commented that “some of the visual language is less clear, people with low literacy would be [confused]” (Interviewer notes of the response of health care professional P156 about message 1A)*.* Another health care professional said the following:

I think it’s highly detailed if you have the time and literacy...but as a general service announcement, I don’t think it’s that effective.

Interviewer notes of the response of health care professional P246 about message 2B

Yet another health care provider said the following:

I think it’s really good but there’s a lot of blocks, which I think someone educated with good eyesight that’s fine, but for someone who is older or low literacy that is too much going on.

Interviewer notes of the response of health care professional P251 about message 1B

For more suggestions and quotes on this topic, see Table 4.

**Table 4:** Public participants’ suggestions for improving the messages with health literacy principles.

Participants’ suggestions	Examples and quotes
Use clear language that is easy to understand; vague terms without definitions are confusing.	Examples of phrases that were confusing: “Reopen intelligently” Vague use of “duration” “When near people, wear a mask” “Forceful exhalation” “Face covering” “High or low occupancy” “Opening intelligently”
Ensure that the “most important” images and messages stand out by making them larger and placing them along the top or top left.	“The mask is a message that needs to be reinforced. If people are going to look at anything, they’ll look at the top row. The middle is busier, so people won’t glance at that, they’ll glance at the top” (Interviewer notes of the response of health care professional P85 about message 1A).
Remove any information that is not strictly necessary to prevent overwhelming viewers.	“What’s really good about this piece is that it puts so much information in one space there is no unnecessary information and it is clear even for people that may not fully understand English” (Interviewer notes of the response of P224 from the Bootheel about message 2A). “I think this one is not as good as the other one. I feel like people are not as likely to really decipher through all the color coding and different info. I feel like the other was more straight forward, direct, easy. This one you have to spend a little more time with it and dig into it” (Interviewer notes of the response of P131 from STL about message 2B).
Ensure that the image is not busy, cluttered, or cramped, and sufficiently space out text and images.	“I feel like it’s too much. They could make it simpler. I can’t even read it, the print is too small. I would need glasses. For example, if this was hung up in a restaurant, I wouldn’t stop to look at it cause it’s just too much, and the print is too small” (Interviewer notes of the response of P269 from the Bootheel about message 1B). “Too info dense; too much wording...given the format it’s cluttered and crowded with too much text” (Interviewer notes of the response of P156, from STL about message 1B).
Colors chosen for the image should enhance the attractiveness and understandability of the message.	“It is a lot more clear because of the colors; [I] suggest a lot more colors and brighter colors so it is more eye-catching” (Interviewer notes of the response of P268 from the Bootheel about message 1B). “It is beautiful for the color which makes it easier to understand” (Interviewer notes of the response of P219 from the Bootheel about message 2B). “It catches your attention, the bright colors draw you in” (Interviewer notes of the response of P15 from STL about message 2A).
People in the images should be diverse (eg, gender, race, and ethnicity) but more realistic looking.	“Better images—use real individuals to be more legible, not every person can like cartoons, real people be better” (Interviewer notes of the response of P146 from STL about message 1A). “I think I’d prefer eyes, nose, and mouth on people. It does look a little funny. I like the diversity of it” (Interviewer notes of the response of P23 from STL about message 1A).
Messages should have emotional appeal to be effective.	“Message like this could appeal more to people’s human nature, something to suggest this is dangerous, people are dying and this is very important, this is informative but doesn’t touch people’s emotions” (Interviewer notes of the response of P05 from STL about message 1A).

### Message Redistribution

On the basis of the survey feedback on our first message sets, we designed new messages to reflect participants’ perspectives. Specifically, we used a list of clear questions rather than directives so that messages could be more readily received and allow readers to make various choices regarding preventive behaviors. We also depicted a diverse (eg, race and ethnicity and age) range of people and activities (eg, eating and outdoor activities) and provided a section on masks that reinforced the main COVID-19 protocols and a link for learning more to establish greater trust with the source. Using the same principles, we also created a message set dedicated to clarifying the postvaccination status. We aimed to reiterate the main COVID-19 protocols [30] and use as little text written in plain language as possible. Our third message set used distinct colors and clear, simple imagery to showcase positive reinforcements for getting vaccinated.

Of the original 65 participants we were able to reach via email (2 participants did not provide an email or gave invalid email addresses), 54 completed the survey, leading to an 83% completion rate. Most participants had an overwhelmingly positive response to the new messages and agreed that the new messages incorporated their feedback from the surveys. Overall, the participants liked the content, bright colors, and simple wording. Common themes expressed by most participants were that they appreciated the simple, precise wording and liked the bright, distinct colors that caught readers’ attention and positive emotional appeal. A participant in St Louis (P23) said that the reminders of what people could do after vaccination “shines” (Interviewer notes of the response of P23 from STL). One of the participants commented the following:

I’m quite impressed by how simple and illustrative the messages are and by just a quick glance I’m able to understand what message the sender wants to portray.

Interviewer notes of the response of P43 from the Bootheel

Another participant said the following:

Yes, [they included my feedback], most certainly so. They made the words larger so everyone can see and also they used more graphic pictures that can be interpreted easily.

Interviewer notes of the response of P225 from the Bootheel in response to the second question in the follow-up survey; full follow-up survey in Multimedia Appendix 3

## Discussion

### Principal Findings

Despite the anticipated differences between the urban and rural populations’ responses to COVID-19 health messages, both groups responded similarly. Both wanted health messages that were consistent, were attractive, were accessible, and emphasized choice in behavioral responses to the pandemic. Furthermore, although our public sample in the Bootheel may have experienced higher COVID-19 exposure and worse social and economic impacts of the pandemic, as indicated by their response to our survey questions on COVID-19, and thus could have had more particular desires for messages owing to personal contexts, the messaging preferences were largely the same between the Bootheel and St Louis samples. This result differs from studies that have found differences between urban and rural populations’ responses to COVID-19 messages [32] and other health messaging campaigns [33]. This may be because both samples had similarly higher levels of health literacy, income, and educational attainment, which may support the participants from both samples to more critically analyze and apply health messages than those with limited health literacy, lower incomes, and lower educational attainment [34]. However, our detailed findings related to people’s similar preferences for the display and content of health messaging might suggest that the socioeconomic, cultural, and political differences between urban and rural communities [8] should not overshadow the development of broadly applicable and well-designed messaging. Although health officials should consider using unique communication channels to reach rural residents [8], such as local newscasters or community health care professionals trusted by the participants in our study, regional differences should not obfuscate the creation of well-designed health messages at the state or national level.

The participants described their preference for COVID-19 protocols to be succinctly presented in each message they saw. They were especially drawn to messaging that called for the use of face masks, social distancing, and other preventive measures. These findings are supported by similar studies conducted in different locations and suggest potentially complex relationships between people and the preventive health behaviors that public health officials, governments, and researchers encourage during health crises. Such actors inconsistently promoted the use of nonpharmaceutical interventions such as masking, and this inconsistency persisted and left members of the public confused on whether masks were advised or which type of mask to wear [35]. Masking may have also emerged as an important and polarizing symbol of the pandemic that had either positive or negative meanings for members of the public [36,37]. Positive resonance with such symbolisms of health interventions could influence people’s reception of the messaging itself. More research is needed to examine people’s relationships with basic preventive health behaviors to help create messaging that can reassure the public and help encourage adherence to such behaviors during periods of uncertainty or rapidly changing safety recommendations [38].

The participants also preferred that personal choice be reflected in COVID-19 messages. That is, they wanted COVID-19 messaging to present the possible repercussions of nonadherence to protocols to inform individuals’ decisions. The importance of personal choice may reflect American beliefs surrounding individual liberties, and messages that appear to infringe on personal freedoms can lead to a decreased likelihood of enacting preventive behaviors [39]. Similar results from a US nationwide poll revealed that words such as “mandates,” “controls,” or “orders” polled lower than the word “protocol” [14]. Other studies have found that philosophical beliefs about liberty may predict an individual’s compliance with public health mandates [40] and that emphasizing individuals’ independence could lead to the adoption of preventive health behaviors [41]. We advise that future health messages be formatted such that they support people in making the best health-related choices for their own lives while also advising effective health prevention behaviors such as masking, especially in the context of participants’ preferences for main COVID-19 protocols. For example, public health officials could disseminate risk indices that display the various risk levels of different settings for readers to determine the best choices for themselves and that explain how and when to wear a mask. This suggestion does not preclude broadcasting necessary health precautions to the general public or the adoption of public health mandates by local, state, and national governments but rather advises altering the tone, word choice, or design to enable personal choice among the various types of preventive behaviors that readers can enact.

The trustworthiness and accessibility of the source of information generated concern among the participants. They wanted to see credible sources and suggested including larger logos for trusted sources, such as the WHO or CDC. They also wanted to see contact information for sources, such as phone numbers or websites. Participants across our samples listed local sources such as friends, family members, local news, physicians, or other health care professionals as their most used sources of information on COVID-19. This finding is consistent with studies that found that facilitating relationships with local stakeholders and health care providers is essential for building trust in COVID-19, especially in rural communities [42,43]. To increase people’s trust in message sources, we recommend including contact information and a specific link to learn more about the health issue as well as using a knowledgeable spokesperson such as a community physician to disseminate new health messages [44]. Furthermore, cobranding health messaging so that local health agencies can share the same information as that shared by national organizations can build trust in populations that have more trust in local sources.

The health care professionals in our study emphasized the need for applicability in COVID-19 messages. They expressed that health messages should be created with the patient populations’ literacy levels and ability to adequately follow the advised protocol in mind. Other studies have suggested the importance of explaining viral spread according to the reader’s level of understanding [3,45]. Our findings demonstrate that consideration of the patient populations is needed for health messages disseminated by health care professionals. We advise that public health officials incorporate feedback from health care professionals when developing health messages and learn more about the specific needs of different patient populations before creating targeted messages.

Participants’ preferences for COVID-19 health messages reflected the best practices for health literacy, emphasizing the importance of these concepts for successful COVID-19 and other health messages. Aspects such as clear communication, prioritization, conciseness, legibility, attractiveness, realism, and emotional appeal were highlighted as essential components for any COVID-19 message [31,46,47]. These qualities resonate strongly with similar studies that found that health messages must have accessible language and clear content [3,13,14]. Incorporating health literacy principles benefits many populations in the United States, including racial or ethnic minorities, groups with lower educational attainment, and those with low socioeconomic status [48,49]. We suggest that designers familiarize themselves with the principles of health literacy [50] and incorporate them into the development of future health messaging. Health literate approaches include using plain language to be concise and conversational [46] and incorporating prosocial messages that emotionally compel readers to comply [51].

Following our initial analysis, the results of which indicated similarities in messaging preferences, we created a short web-based survey for original participants to comment on new messages created based on their original surveys, continuing participants’ engagement in the research process. Our high completion rate for the survey on message redistribution and participants’ appreciation for the incorporation of their feedback from the initial surveys indicate the importance of continued contact with research participants. Disseminating results back to participants and engaging them throughout the message development process can improve the trust in researchers and strengthen the ties between research organizations and various communities. Other studies have found that creative methods of recontacting participants and disseminating results in the form of community listening sessions or research forums can improve the willingness to participate in research [52,53]. Building on this literature, short web-based surveys and community-based message creation may add to the data collection methods that health literacy researchers can use when attempting to engage participants in the research process. Such web-based methods have the benefit of being more accessible and less resource intensive and time consuming than other research methods [54].

Informed by our findings, we created refined health messaging that incorporated the themes participants discussed during their surveys to disseminate examples of health messaging that both incorporated participants’ varied preferences and aligned with health communication best practices. These messages were action oriented and uniquely addressed personal choice in health prevention, common health protocols, and accessible source information. We used a list of questions to prompt readers to consider their risk when planning activities to present less overwhelming visual content and align with participants’ preferences for personal choice. When communicating complex topics, such as personal risk and probabilities, researchers and public health officials often use visual depictions, such as icon arrays and figures, to help enhance the understanding of numerical estimates [55]. However, high amount of numerical information has the potential to overwhelm viewers, especially those with limited overall literacy or health literacy [56-58]. Future risk messaging might consider using gist representations of risk to inspire readers to consider the general magnitude of their risk [55]. For those seeking more precise, verbatim risk information, links or QR codes can provide more detailed probabilistic information. Incorporating numeric information that is easy to understand can guide the development of engaging and useful health messages.

### Limitations, Strengths, and Future Directions

This study has multiple limitations and strengths that indicate potential avenues for future research on people’s opinions related to health messaging. First, we used self-selection methods for recruitment, which may have attracted individuals who were highly motivated to participate in a study related to COVID-19. These methods may have also resulted in samples of people with higher incomes, educational attainment, and health literacy scores than the general public in both St Louis and the Bootheel. At the time of data collection, which was during the early phases of the COVID-19 pandemic, remote recruitment based on self-selection was our only recruitment option, which likely limited the populations we were able to reach for our study. These methods may also have resulted in samples of people with higher health literacy scores, incomes, and educational attainment than the general public in both St Louis and the Bootheel. Such selection bias may suggest that participants were more predisposed to respond positively to COVID-19 mitigation efforts and express preferences for messaging that suggested behavioral interventions for COVID-19 spread. Difficulties in recruiting health care professionals in the Bootheel likely arose because of the overall lack of providers in the area and the strained schedules of providers during the time of the study. Future research can use different recruitment methods to gather a more representative sample of urban and rural regions to adequately examine the nuances in regional responses to health messaging.

Potentially owing to our sampling methods, our results differ from other findings of rural populations’ hesitance and distrust toward behavioral recommendations related to COVID-19 [10,11]. However, our findings may still resonate with other studies that document that even though rural populations are less likely to participate in preventive health behaviors related to COVID-19, they may still highly believe in the efficacy of public health measures and the threat of the pandemic to their community and be open to receiving health messages from trusted local health officials [59,60]. Our findings may also indicate that it is important that researchers not homogenize rural populations’ approaches to the pandemic and instead dedicate more resources to addressing how rural populations understand their health. Furthermore, although we did not evaluate participants’ level of understanding of the health messages, we know that the mastery of what people attend to in health messages is vital in how we design and distribute health messages and inform the public. Future research can evaluate whether disseminating appealing public health messages translates into the comprehension of the message content. Moreover, we recruited participants from a Midwestern state in the United States, meaning that the results may not be applicable to other geographic areas, and our samples did not include racial and ethnic groups that were not White or Black. However, our mixed methods approach and thematic analysis revealed areas of improvement that can strengthen public health messaging and reinforce the importance of best practices for effective health messaging.

In addition, although our data represent participant perspectives from a relatively early point in the pandemic, the message redistribution method may continue to prove useful when examining other health literacy issues in the context of urban and rural health disparities. These disparities continue to be observed in cancer prevalence [61], cardiovascular care [62], and other health domains. More research is required to fully examine local contexts and attitudes toward COVID-19 messaging, but our findings can improve and inform public health messaging so that it is as clear, applicable, and effective as possible.

### Conclusions

This analysis of participants’ responses indicates areas of improvement for future health messaging, such as reaffirming common COVID-19 protocols, framing content such that it allows for personal choice, and advertising easily accessible source information. Messages communicated by health care professionals should align with the needs of specific patient populations, and all messages must include plain language, effective wording, emotional appeal, and an attractive design. Participants’ engagement in message creation can aid in health equity implementation. These findings are critical for stakeholders developing public health messages for the COVID-19 pandemic and other public health crises.

## Appendix

Multimedia Appendix 1 The survey given to the members of the public samples during this study.

Multimedia Appendix 2 The survey used for the providers in our study.

Multimedia Appendix 3 The messages we created in response to participant feedback as well as the brief survey we used to collect participants’ opinions on the newly created messages.

## Abbreviations

CDC: Centers for Disease Control and Prevention
HIPAA: Health Insurance Portability and Accountability Act
REDCap: Research Electronic Data Capture
STL: St Louis metropolitan region
WHO: World Health Organization

## Data Availability

Data are available upon reasonable request to the corresponding author. Data are not publicly available to protect the confidentiality of the study participants.
