# Supplementary material for: Eliciting Opinions on Health Messaging During the COVID-19 Pandemic: Qualitative Survey Study
Source: JMIR Hum Factors. 2023 Apr 27;10:e39697. doi: 10.2196/39697 (PMC10176135; doi:10.2196/39697)
Supplement: Multimedia Appendix 1 [file humanfactors_v10i1e39697_app1.docx]

**Appendix 1 – The survey given to the members of the public samples during this study.**

Thank you for agreeing to talk with us today. We’re going to start with some questions about your thoughts about COVID-19.

1. How much attention would you say you have paid to COVID-19?

___A lot

___Some

___A little

1. How much does COVID-19 affect your life?

___ Has a major affect

___ Has a moderate affect

___ Has a minor affect

___ Has no affect

1. How much control do you feel you have over whether or not you get COVID-19?

___ Complete control

___ Some control

___ A little control

___ No control

1. How much do you think treatment can help people who get COVID-19?

___ A great deal

___ Some

___ A little

___ Not at all

1. How much does COVID-19 affect you emotionally (e.g. how much do you feel angry, scared, upset, worried, or depressed)?

___ A great deal

___ Somewhat

___ A little

___ Not at all

1. Have you ever been tested for COVID-19?

___Yes – When? _____________

___No

___Not sure/ don’t know

___Prefer not to answer

1. Have you ever been diagnosed with COVID-19?

___Yes – When? _____________

___No

___Not sure/ don’t know

___Prefer not to answer

1. Have you ever thought you might have COVID-19 but didn’t go to a doctor to have a test?

___Yes – When? _____________

___No

___Not sure/ don’t know

___Prefer not to answer

1. Has anyone in your family been tested for COVID-19?

___Yes – When? _____________

___No

___Not sure/ don’t know

___Prefer not to answer

1. Has anyone in your family thought they might have had COVID-19 but didn’t go to a doctor to have a test?

___Yes – When? _____________

___No

___Not sure/ don’t know

___Prefer not to answer

1. Has anyone close to you tested positive for COVID-19?

___Yes – When? _____________

___No

___Not sure/ don’t know

___Prefer not to answer

11b. If any yes, do they live in the same home you live in?

___Yes

___No

1. How many people do you know who have had COVID-19?

___None

___1

___2-5

___6 or more

1. Do you know anyone who has been hospitalized for COVID-19?

___Yes – When? _____________

___No

___Not sure/ don’t know

___Prefer not to answer

13a. If any yes, do they live in the same home you live in?

___Yes

___No

1. Do you know anyone who has died from COVID-19?

___Yes – When? _____________

What was your relation to that person? _____________

___No

___Not sure/ don’t know

___Prefer not to answer

1. On a scale from 1 to 10, where 1 means you are not worried at all and 10 means you are extremely worried…

|  | Not worried at all | Somewhat worried | Extremely worried |
| --- | --- | --- | --- |
| How worried have you been about getting COVID-19? | 1 2 3 4 5 6 7 8 9 10 | | |
| How worried are you that others around you will get COVID-19? | 1 2 3 4 5 6 7 8 9 10 | | |
| How worried have you been about not being able to afford or access food because of the COVID-19 outbreak? | 1 2 3 4 5 6 7 8 9 10 | | |
| How worried have you been about access to important resources such as transportation or housing due to the COVID-19 outbreak? | 1 2 3 4 5 6 7 8 9 10 | | |

1. From 1 to 10, where 1 means not at all and 10 means extremely…

|  | Not at all | Somewhat | Extremely |
| --- | --- | --- | --- |
| To what degree has the COVID-19 pandemic in your area created financial problems for you or your family | 1 2 3 4 5 6 7 8 9 10 | | |

1. How much of the day do you actively seek information about COVID-19?

___ Not at all

___ About 1 hour

___ 2-3 hours

___ 4-6 hours

___ More than 6 hours

1. How great of a threat do you feel COVID-19 poses to your family?

___ A great threat

___ A moderate threat

___ Some threat

___ No threat

1. How great of a threat do you feel COVID-19 poses to your community?

___ A great threat

___ A moderate threat

___ Some threat

___ No threat

1. In your opinion, how effective are the following actions for keeping you safe from COVID-19?

|  | Not effective at all | Hardly effective | Somewhat effective | Effective | Very effective |
| --- | --- | --- | --- | --- | --- |
| Wearing a face mask | 0 | 1 | 2 | 3 | 4 |
| Praying | 0 | 1 | 2 | 3 | 4 |
| Washing your hands with soap or using hand sanitizer frequently | 0 | 1 | 2 | 3 | 4 |
| Seeing a health care provider if you feel sick | 0 | 1 | 2 | 3 | 4 |
| Seeing a health care provider if you feel healthy buy worry that you were exposed | 0 | 1 | 2 | 3 | 4 |
| Avoiding public spaces, gatherings, and crowds | 0 | 1 | 2 | 3 | 4 |
| Avoiding contact with people who could be high-risk | 0 | 1 | 2 | 3 | 4 |
| Avoiding hospitals and clinics | 0 | 1 | 2 | 3 | 4 |
| Avoiding restaurants | 0 | 1 | 2 | 3 | 4 |
| Avoiding public transport | 0 | 1 | 2 | 3 | 4 |

1. Please tell me if you do the following things more, less, or about the same as you did before the COVID-19 pandemic

|  | Do this less than before | Do this about the same as before | Do this more than before |
| --- | --- | --- | --- |
| Amount you sleep | 1 | 2 | 3 |
| Amount you smoke or vape | 1 | 2 | 3 |
| Amount of alcohol you drink | 1 | 2 | 3 |
| Number of hours you work in your usual workplace | 1 | 2 | 3 |
| Number of hours you work from home | 1 | 2 | 3 |
| Time spent talking to family/friends inside your home | 1 | 2 | 3 |
| Time spent talking to family/friends outside your home (over the phone or zoom) | 1 | 2 | 3 |
| Time spent talking to work colleagues | 1 | 2 | 3 |
| Practicing relaxation/ mindfulness/meditation | 1 | 2 | 3 |
| Time spent listening to the news on radio or TV | 1 | 2 | 3 |
| Time spent using other devices with a screen | 1 | 2 | 3 |
| Time spent doing hobbies/things you enjoy | 1 | 2 | 3 |
| Amount of money you’ve spent | 1 | 2 | 3 |
| Amount of physical activity/exercise you do | 1 | 2 | 3 |
| Time spent travelling on public transport | 1 | 2 | 3 |
| Time spent travelling in a car | 1 | 2 | 3 |
| Time spent travelling on a bike | 1 | 2 | 3 |
| Time spent outdoors | 1 | 2 | 3 |

1. Tell me if you think the following statements are true or false…

|  | **False** | **True** |
| --- | --- | --- |
| People exposed to COVID-19 can spread the disease to others, even if they do not have any symptoms. | 0 | 1 |
| The symptoms of COVID-19 are connected to 5G mobile network radiation. | 0 | 1 |
| Regularly washing your hands for 20 seconds can help protect you from COVID-19. | 0 | 1 |

1. Tell me which of the following sources you have used to get information about COVID-19.

|  | **No** | **Yes** |
| --- | --- | --- |
| Twitter | 0 | 1 |
| Facebook | 0 | 1 |
| Newspaper | 0 | 1 |
| Friends or family members | 0 | 1 |
| Coworkers or classmates | 0 | 1 |
| Doctors or other health care providers | 0 | 1 |
| Official government websites | 0 | 1 |
| President Trump | 0 | 1 |
| State Governor/Mayor | 0 | 1 |
| World Health Organization (WHO) | 0 | 1 |
| Centers for Disease Control (CDC) | 0 | 1 |
| State, County, or City health department | 0 | 1 |
| CNN | 0 | 1 |
| Fox News | 0 | 1 |
| MSNBC | 0 | 1 |
| Local news station (e.g. CBS, ABC, NBC) | 0 | 1 |
| National Public Radio (NPR) | 0 | 1 |

1. Of those sources, which do you use the most to learn about COVID-19? _____________
2. Of those sources, which do you use the least to learn about COVID-19? _____________
3. To what extent are you avoiding information about COVID-19? Please tell me on a scale, again, from 1 to 10 where 1 means you are seeking information and 10 means you are always trying to avoid information about the virus. CIRCLE RESPONSE.

| **Seeking Information** |  |  |  | **Not seeking or avoiding information** |  |  |  |  | **Always trying to avoid information** |
| --- | --- | --- | --- | --- | --- | --- | --- | --- | --- |
| 1 | 2 | 3 | 4 | 5 | 6 | 7 | 8 | 9 | 10 |

1. How long do you think the COVID-19 pandemic will last? ______________________________
2. Do you think there will be a vaccine for COVID-19?

___Yes

___No

___Not sure/ don’t know

1. If there is a vaccine for COVID-19, will you get the vaccine?

___Yes

___No

___Not sure/ don’t know

1. What are the three most important things that you believe caused COVID-19 to appear in the world?

1.____________________________________________________________

2.____________________________________________________________

3.____________________________________________________________

1. Now, what are the three most important things that you believe caused COVID-19 to appear where you live?

1.____________________________________________________________

2.____________________________________________________________

3.____________________________________________________________

1. Could you explain to me the ways COVID-19 spreads between people?

PROBE – What can people do to stop that spread?

PROBE – Have you heard of anything people are talking about doing that won’t help stop that spread? What was that?

1. How has COVID-19 impacted your life?
2. Based on what you have experienced or seen so far, what advice would you have for others about COVID-19?

**- - - - - - - - - - - - - - - - - - - - - - - - - - - - - - - - - - - - - - - - - - - - - -**

Now, we’d like to show you 2 examples of communication about COVID-19 and get your responses to them. You have them on the hand-out we sent you earlier.

Let’s look at the first example of communication about COVID-19. Tell me when you think you have a good understanding of this.

1. What is your overall impression of the piece?
2. What are the most and least important messages?

MOST

LEAST

1. Do you think anything is missing in this message? If so, what?
   1. If don’t know/not sure – what makes it hard to decide?
2. Is there anything in this that you don’t fully understand?
   1. If so, what?
   2. If don’t know/not sure – what makes it hard to decide?
3. Is there anything in this that your friends and family wouldn’t understand?
   1. If yes, what would they not understand?
   2. If don’t know/not sure – what makes it hard to decide?
4. How do you feel about the overall design and look of the piece?

PROBE --If you could help the designer of the piece, what would you suggest might be changed to improve it?

1. These type of graphics – sometimes called infographics – are meant to help people make decisions about their life. How does the information in this piece match up to how you are living through COVID-19?

PROBE – What, if anything, in this piece might lead you to change something about your life?

PROBE – (IF nothing) … why wouldn’t you change your decisions or behavior based on this information?

PROBE - If don’t know/not sure – what makes it hard to decide?

1. How do you think your friends would react to this?
2. What information in this do you not believe?
3. What information in this do you think will change in the future?

PROBE - Why do you say that?

1. How likely are you to go to the website or source provided to find out more?

PROBE - Why do you say that?

1. Is there anything else you’d like to say about this image and the messages it contains?

**- - - - - - - - - - - - - - - - - - - - - - - - - - - - - - - - - - - - - - - - - - - - - -**

OK, we have just a few more questions that will help us know more about you and others who participated in this research. These will help us in our analysis but will not identify you
in anyway.

1. What is your age? _______ years
2. What is your gender?

___Female

___Male

___Non-binary

___Prefer to self-describe as __________________________

___Prefer not to say

1. How many years of formal education have you completed? _____ years

___ 8^th^ grade or less

___ Some high school

___ Graduated high school or GED

___ Vocational School

___ Associate degree

___ Bachelor’s degree

___ Graduate or professional degree

1. What is your employment status?

___Full time

___Part time

___Retired

___Unemployed

1. Do you have more than one job?

___Yes

___No

___If yes, total # of Jobs_____________________

1. Are you what is considered an essential worker?

___Yes

___No

___Don’t know/not sure

52b. If so, are you provided protective equipment (e.g. masks, gloves, face shields) at work?

___Yes

___No

___Don’t know/not sure

1. Does someone else who is considered an essential worker live in your home?

___Yes

___No

___Don’t know/not sure

53b. If yes, is that person provided protective equipment at work?

___Yes

___No

___Don’t know/not sure

1. How often do you use any form of public mass transportation?

___ Most of the time

___ Some of the time

___ Seldom

___ Never

1. What’s your yearly family income, including all sources?

___ Less than $15,000

___ $15,000-$34,999

___$35,000-$54,999

___$55,000-$74,999

___$75,000 or more

___Prefer not to answer

1. Please tell me about your health insurance?

___Medicaid

___Medicare

___Employer-based insurance

___Tri-Care

___Union-based

___Self pay (purchase insurance on own)

___Other________________

___Uninsured (no insurance)

1. How many children do you have? Either living at home or not.

______________

1. How many people currently live in your home?

______________

1. Please tell us your race or ethnicity

___ White ___ African American or Black

___ Asian or Pacific Islander ___ American Indian or Alaska Native

___ Hispanic or Latino ___ Other, specify ___________________

1. Primary Language spoken at home:

___ English

___ Spanish

___ Other:______________________________________

1. I would like to ask you some questions about your experiences with your neighbors and in your neighborhood. Please tell me whether you agree or disagree with the following statements.

|  | Disagree | Agree |
| --- | --- | --- |
| I can count on people in my neighborhood to help me if I’m sick | 0 | 1 |
| My neighbors would go to the store for me if I’m sick | 0 | 1 |

For this question, please try to answer using Never, Sometimes, Often, or Always to tell us how often you do each activity.

1. How often do you do each task below?

|  | Never | Sometimes | Often | Always |
| --- | --- | --- | --- | --- |
| Find or look for health information | 0 | 1 | 2 | 3 |
| Understand information about  your health | 0 | 1 | 2 | 3 |
| Evaluate how health information  relates to your life (If asked --- for  example, decide if and how information  is relevant to your life) | 0 | 1 | 2 | 3 |
| Communicate about your health  to others | 0 | 1 | 2 | 3 |
| Act on information about your health | 0 | 1 | 2 | 3 |

1. Would you say that in general your health is…?

___Excellent

___Very good

___Good

___Fair

___Poor

1. Thinking about your physical health, which includes physical illness and injury, for how many days during the past 30 days was your physical health not good?

___Number of days

1. Now thinking about your mental health, which includes stress, depression, and problems with emotions, for how many days during the past 30 days was your mental health not good?

___Number of days

1. Do you get a flu shot every year?

___Yes

___No

| 1. PHQ-9 Depression Scale | | | | |
| --- | --- | --- | --- | --- |
|  | Not at all | Several days | More than half the days | Nearly every day |
| Over the last 2 weeks, how often have you been bothered by any of the following problems?  Read each item carefully, and circle the response. | | | | |
| Little interest or pleasure in doing things | 0 | 1 | 2 | 3 |
| Feeling down, depressed, or hopeless | 0 | 1 | 2 | 3 |
| Trouble falling asleep, staying asleep, or sleeping too much | 0 | 1 | 2 | 3 |
| Feeling tired or having little energy | 0 | 1 | 2 | 3 |
| Poor appetite or overeating | 0 | 1 | 2 | 3 |
| Feeling bad about yourself, feeling that you are a failure, or feeling that you have let yourself or your family down | 0 | 1 | 2 | 3 |
| Trouble concentrating on things such as reading the newspaper or watching television | 0 | 1 | 2 | 3 |
| Moving or speaking so slowly that other people could have noticed. Or being so fidgety or restless that you have been moving around a lot more than usual | 0 | 1 | 2 | 3 |
| Thinking that you would be better off dead or that you want to hurt yourself in some way  If 2 or 3:  How are you feeling currently? Would you like some  mental health resources? __________________________________  If you currently feel that you are having acute mental health problems, call the Behavioral Health Response ACI Hotline at 314-469-6644 or 800-811-4760. We can also stop the interview here if you'd like. | 0 | 1 | 2 | 3 |

|  | Not difficult at all | Somewhat difficult | Very difficult | Extremely Difficult |
| --- | --- | --- | --- | --- |
| Description of difficulty |  |  |  |  |
| If response is 1, 2, or 3 on any of the items above: How difficult have these problems made it for you to do your work, take care of things at home, or get along with other people?  If 2 or 3:  How are you feeling currently? Would you like some  mental health resources? __________________________________  If you currently feel that you are having acute mental health problems, call the Behavioral Health Response ACI Hotline at 314-469-6644 or 800-811-4760. We can also stop the interview here if you'd like. | 0 | 1 | 2 | 3 |

1. Have you ever been diagnosed with any of the following chronic diseases? (MARK ALL THAT APPLY)

___ Asthma

___ Heart Disease (CVD)

___ Diabetes

___ Depression

___ Hypertension (high blood pressure)

___ Overweight/obesity

___ High Cholesterol

___ Any others________________________

1. Do you currently or have you ever smoked any tobacco products?

___ Yes, now

___ Used to but not now

___ Never have

1. For current smokers only: On average how many of the following products do you currently smoke each day?

Manufactured cigarettes (# of cigarettes, not packs) _____/day

Hand-rolled cigarettes? _____/day

Kreteks? _____/day

Pipes full of tobacco? _____/day

Cigars, cheroots, or cigarillos? _____/day

Water pipe sessions? _____/day

Any others? _____/day – Please specify what ______________________________

That’s all the questions I have for you today. Is there anything you think would be important to discuss that we haven’t already covered?
