# Supplementary material for: Eliciting Opinions on Health Messaging During the COVID-19 Pandemic: Qualitative Survey Study
Source: JMIR Hum Factors. 2023 Apr 27;10:e39697. doi: 10.2196/39697 (PMC10176135; doi:10.2196/39697)
Supplement: Multimedia Appendix 2 [file humanfactors_v10i1e39697_app2.docx]

**Appendix 2 – Healthcare Professional Survey**

Thank you for agreeing to talk with us today. We’re going to start with some questions about your thoughts about COVID-19.

1. How much attention would you say you have paid to COVID-19?

___A lot

___Some

___A little

1. How much does COVID-19 affect your life?

___ Has a major affect

___ Has a moderate affect

___ Has a minor affect

___ Has no affect

1. How much do you think treatment can help people who get COVID-19?

___ A great deal

___ Some

___ A little

___ Not at all

1. How much does COVID-19 affect you emotionally (e.g. how much do you feel angry, scared, upset, worried, or depressed)?

___ A great deal

___ Somewhat

___ A little

___ Not at all

1. How much of the day do you actively seek information about COVID-19?

___ Not at all

___ About 1 hour

___ 2-3 hours

___ 4-6 hours

___ More than 6 hours

1. How great of a threat do you feel COVID-19 poses to your patients?

___ A great threat

___ A moderate threat

___ Some threat

___ No threat

1. How great of a threat do you feel COVID-19 poses to your community?

___ A great threat

___ A moderate threat

___ Some threat

___ No threat

1. In your opinion, how effective are the following actions for keeping people safe from COVID-19?

|  | Not effective at all | Hardly effective | Somewhat effective | Effective | Very effective |
| --- | --- | --- | --- | --- | --- |
| Wearing a face mask | 0 | 1 | 2 | 3 | 4 |
| Praying | 0 | 1 | 2 | 3 | 4 |
| Washing your hands with soap or using hand sanitizer frequently | 0 | 1 | 2 | 3 | 4 |
| Seeing a health care provider if you feel sick | 0 | 1 | 2 | 3 | 4 |
| Seeing a health care provider if you feel healthy buy worry that you were exposed | 0 | 1 | 2 | 3 | 4 |
| Avoiding public spaces, gatherings, and crowds | 0 | 1 | 2 | 3 | 4 |
| Avoiding contact with people who could be high-risk | 0 | 1 | 2 | 3 | 4 |
| Avoiding hospitals and clinics | 0 | 1 | 2 | 3 | 4 |
| Avoiding restaurants | 0 | 1 | 2 | 3 | 4 |
| Avoiding public transport | 0 | 1 | 2 | 3 | 4 |

1. Please tell me if your patients describe doing the following things more, less, or about the same as they did before the COVID-19 pandemic.

|  | Patients report doing this less than before | Patients report doing this about the same as before | Patients report doing this more than before | Patients have not discussed |
| --- | --- | --- | --- | --- |
| Amount they sleep | 1 | 2 | 3 | 4 |
| Amount they smoke or vape | 1 | 2 | 3 | 4 |
| Amount of alcohol they drink | 1 | 2 | 3 | 4 |
| Number of hours they work in their usual workplace | 1 | 2 | 3 | 4 |
| Number of hours they work from home | 1 | 2 | 3 | 4 |
| Time spent talking to family/friends inside their home | 1 | 2 | 3 | 4 |
| Time spent talking to family/friends outside their home (over the phone or Zoom) | 1 | 2 | 3 | 4 |
| Time spent talking to work colleagues | 1 | 2 | 3 | 4 |
| Practicing relaxation/ mindfulness/meditation | 1 | 2 | 3 | 4 |
| Time spent listening to the news on radio or TV | 1 | 2 | 3 | 4 |
| Time spent using other devices with a screen | 1 | 2 | 3 | 4 |
| Time spent doing hobbies/things they enjoy | 1 | 2 | 3 | 4 |
| Amount of money they spend | 1 | 2 | 3 | 4 |
| Amount of physical activity/exercise they do | 1 | 2 | 3 | 4 |
| Time spent travelling on public transport | 1 | 2 | 3 | 4 |
| Time spent travelling in a car | 1 | 2 | 3 | 4 |
| Time spent travelling on a bike | 1 | 2 | 3 | 4 |
| Time spent outdoors | 1 | 2 | 3 | 4 |

1. Tell me which of the following sources you have used to get information about COVID-19.

|  | **No** | **Yes** |
| --- | --- | --- |
| Twitter | 0 | 1 |
| Facebook | 0 | 1 |
| Newspaper | 0 | 1 |
| Friends or family members | 0 | 1 |
| Coworkers or classmates | 0 | 1 |
| Doctors or other health care providers | 0 | 1 |
| Official government websites | 0 | 1 |
| President Trump | 0 | 1 |
| State Governor/Mayor | 0 | 1 |
| World Health Organization (WHO) | 0 | 1 |
| Centers for Disease Control (CDC) | 0 | 1 |
| State, County, or City health department | 0 | 1 |
| CNN | 0 | 1 |
| Fox News | 0 | 1 |
| MSNBC | 0 | 1 |
| Local news station (e.g. CBS, ABC, NBC) | 0 | 1 |
| National Public Radio (NPR) | 0 | 1 |

1. Of those sources, which do you use the most to learn about COVID-19? _____________
2. Of those sources, which do you use the least to learn about COVID-19? _____________
3. How long do you think the COVID-19 pandemic will last? ______________________________
4. Do you think there will be a vaccine for COVID-19?

___Yes

___No

___Not sure/ don’t know

1. If there is a vaccine for COVID-19, will you recommend the vaccine to your patients?

___Yes

___No

___Not sure/ don’t know

- 1. If yes: If your patient was hesitant to get the vaccine, how would you respond?
  2. If no or not sure: Why?
  3. If no or not sure: What would you tell patients who request the vaccine?

1. How do you explain to your patients the ways COVID-19 spreads between people?

PROBE – Have you heard of anything people are talking about doing that won’t help stop that spread? What was that?

1. Based on what you have experienced or seen so far, what advice would you have for other medical professionals?

**- - - - - - - - - - - - - - - - - - - - - - - - - - - - - - - - - - - - - - - - - - - - - -**

Now, we’d like to show you 2 examples of communication about COVID-19 and get your responses to them. You have them on the hand-out we sent you earlier.

Let’s look at the first example of communication about COVID-19. Tell me when you’re done reviewing it.

1. What is your overall impression of the piece?
2. What are the most and least important messages?

MOST

LEAST

1. Do you think anything is missing in this message? If so, what?
   1. If don’t know/not sure – what makes it hard to decide?
2. Is there anything in this that you think your patients wouldn’t fully understand?
   1. If so, what?
   2. If don’t know/not sure – what makes it hard to decide?
3. How do you feel about the overall design and look of the piece?

PROBE --If you could help the designer of the piece, what would you suggest might be changed to improve it?

1. These type of graphics – sometimes called infographics – are meant to help people make decisions about their life. How does the information in this piece match up to how you are talking to your patients about COVID-19?
2. What information in this do you think will change in the future?

PROBE - Why do you say that?

1. Is there anything else you’d like to say about this image and the messages it contains?

**- - - - - - - - - - - - - - - - - - - - - - - - - - - - - - - - - - - - - - - - - - - - - -**

OK, we have just a few more questions that will help us know more about you and others who participated in this research. These will help us in our analysis but will not identify you
in anyway.

1. What is your age? _______ years
2. What is your gender?

___Female

___Male

___Non-binary

___Prefer to self-describe as __________________________

___Prefer not to say

1. What is your professional degree?
2. How many years have you been in practice?
3. What is your employment status?

___Full time

___Part time

___Retired

___Unemployed

1. In what setting do you work (e.g., primary care office, emergency department)?

31b. Are you provided sufficient protective equipment (e.g. masks, gloves, face shields) at work?

___Yes

___No

___Don’t know/not sure

1. What health insurance does your workplace accept?

___Medicaid

___Medicare

___Employer-based insurance

___Tri-Care

___Union-based

___Self pay (purchase insurance on own)

___Other________________

___Uninsured (no insurance)

1. Please tell us your race or ethnicity

___ White ___ African American or Black

___ Asian or Pacific Islander ___ American Indian or Alaska Native

___ Hispanic or Latino ___ Other, specify ___________________

1. What is the primary language for most of your patients?

___ English

___ Spanish

___ Other:______________________________________

1. Thinking about your mental health, which includes stress, depression, and problems with emotions, for how many days during the past 30 days was your mental health not good?

___Number of days

1. Do you get a flu shot every year?

___Yes

___No

That’s all the questions I have for you today. Is there anything you think would be important to discuss that we haven’t already covered?
