# Supplementary material for: Eliciting Opinions on Health Messaging During the COVID-19 Pandemic: Qualitative Survey Study
Source: JMIR Hum Factors. 2023 Apr 27;10:e39697. doi: 10.2196/39697 (PMC10176135; doi:10.2196/39697)
Supplement: Multimedia Appendix 3 [file humanfactors_v10i1e39697_app3.docx]

**Appendix 3 – Newly Created Messages and Message Redistribution Survey**


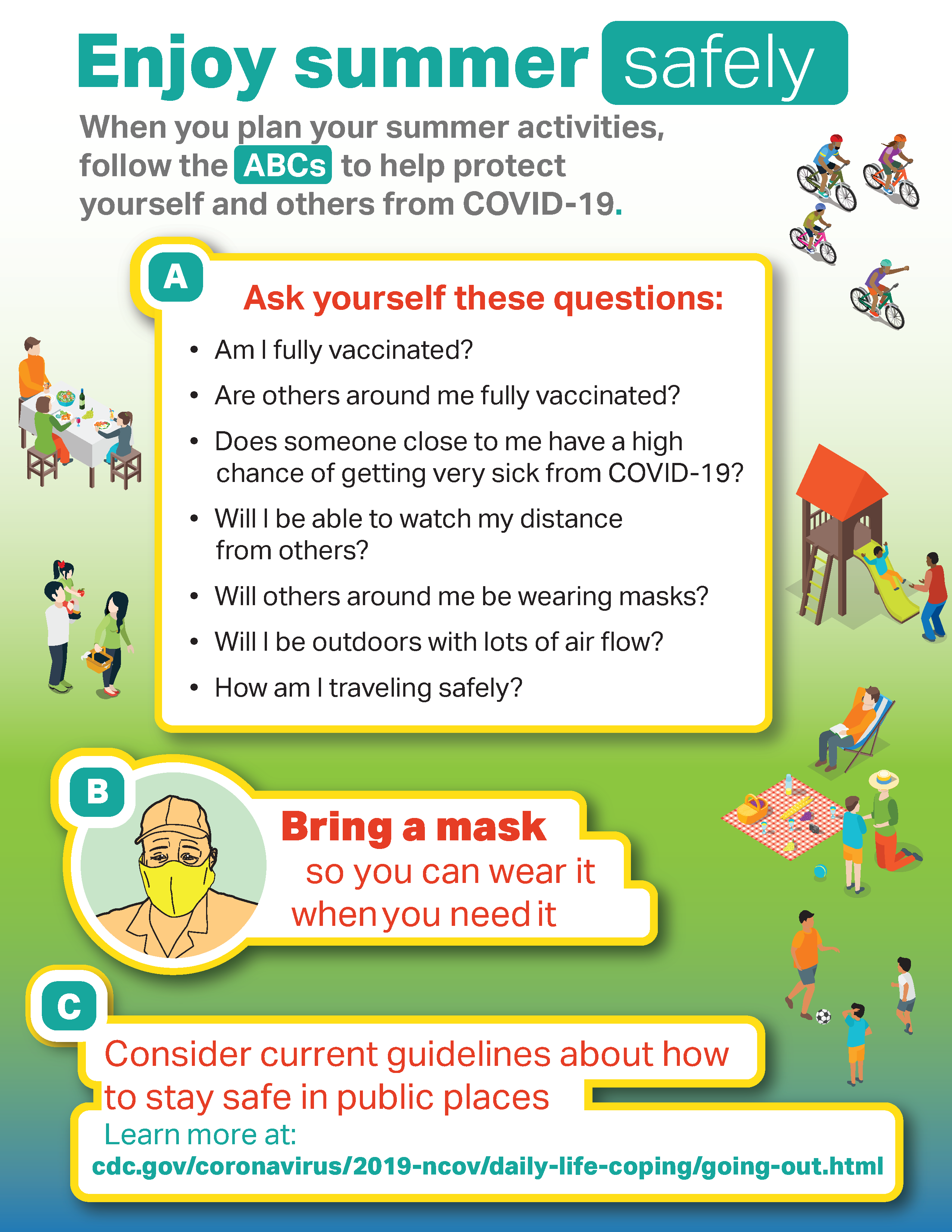


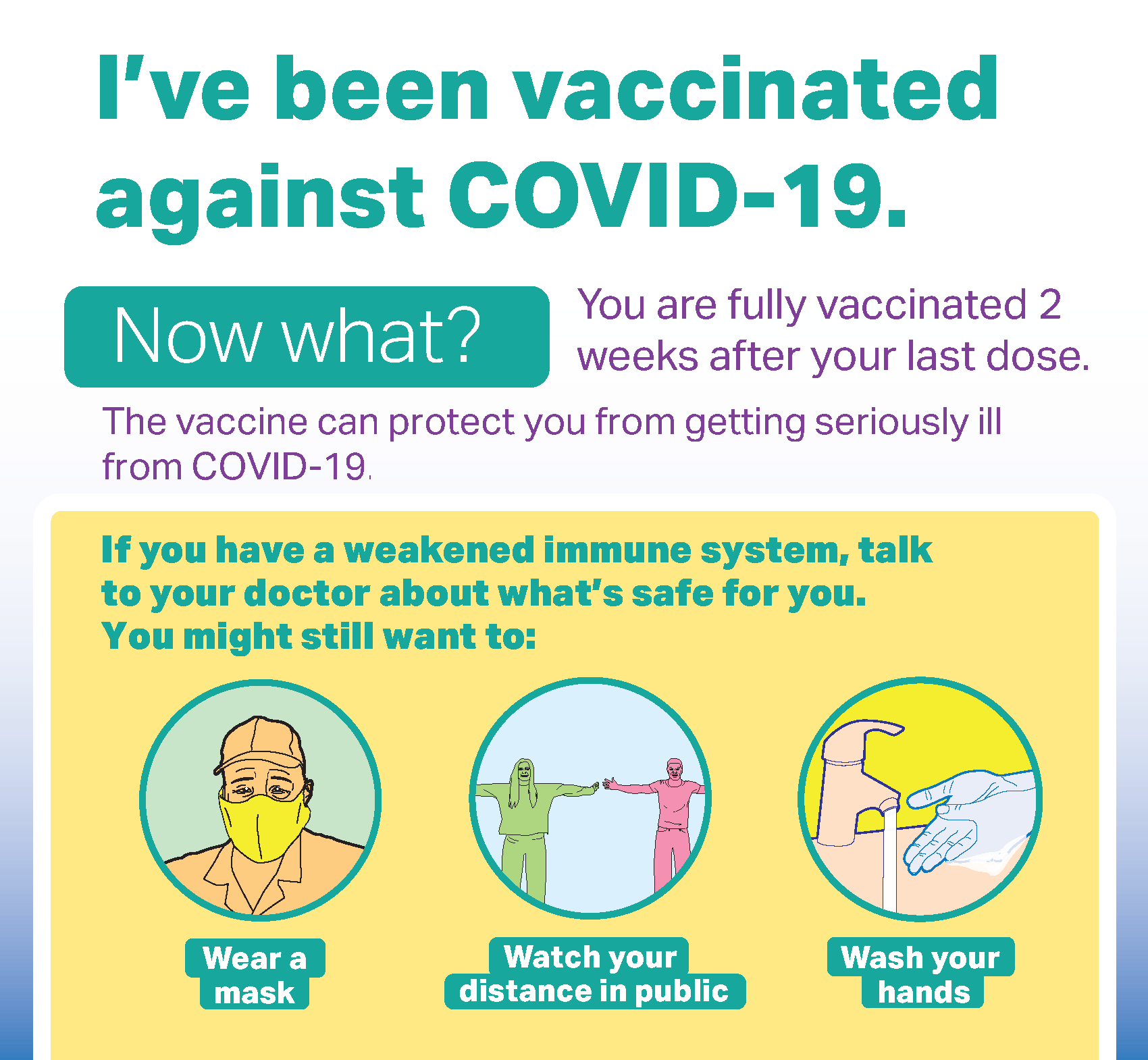


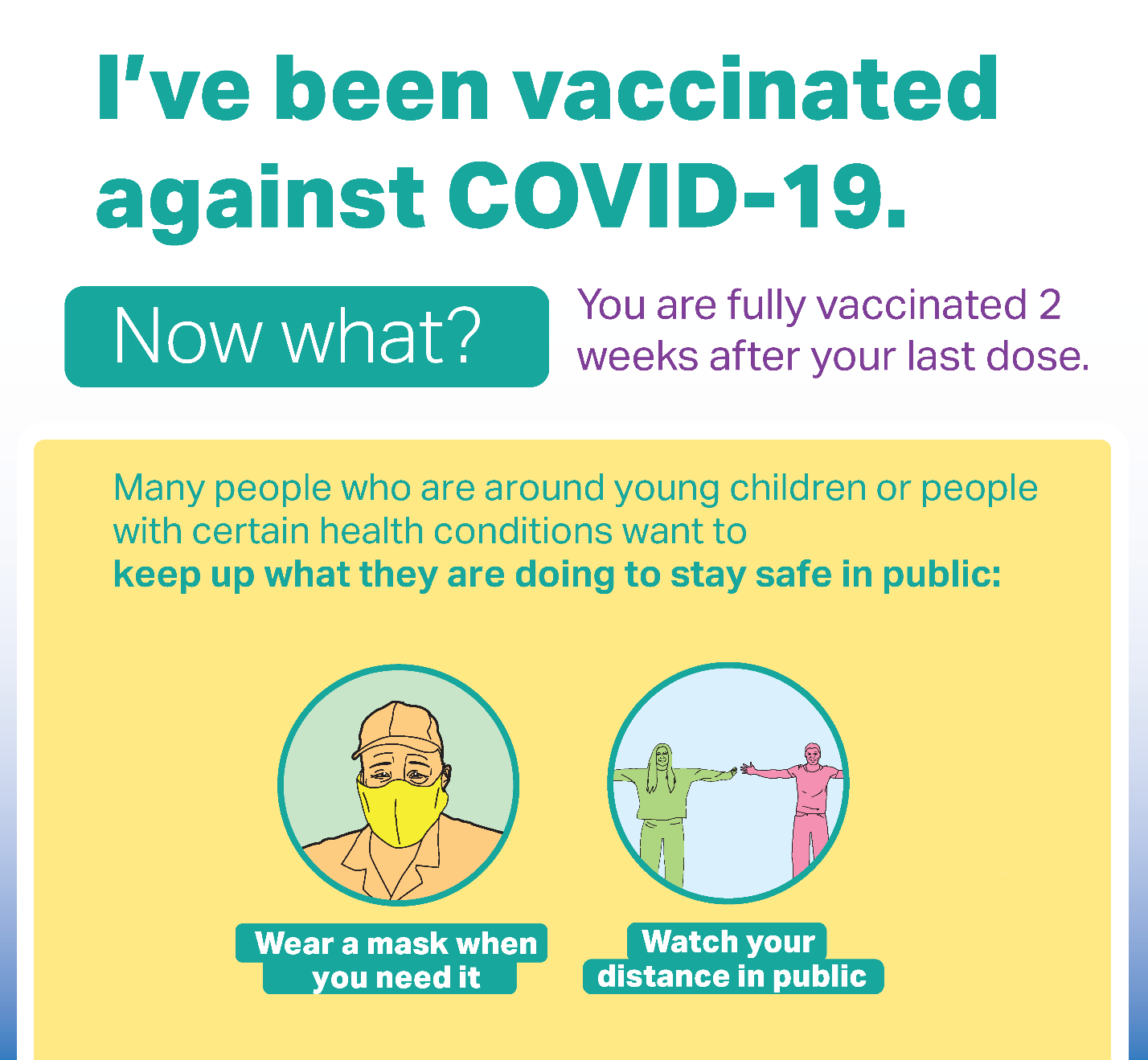


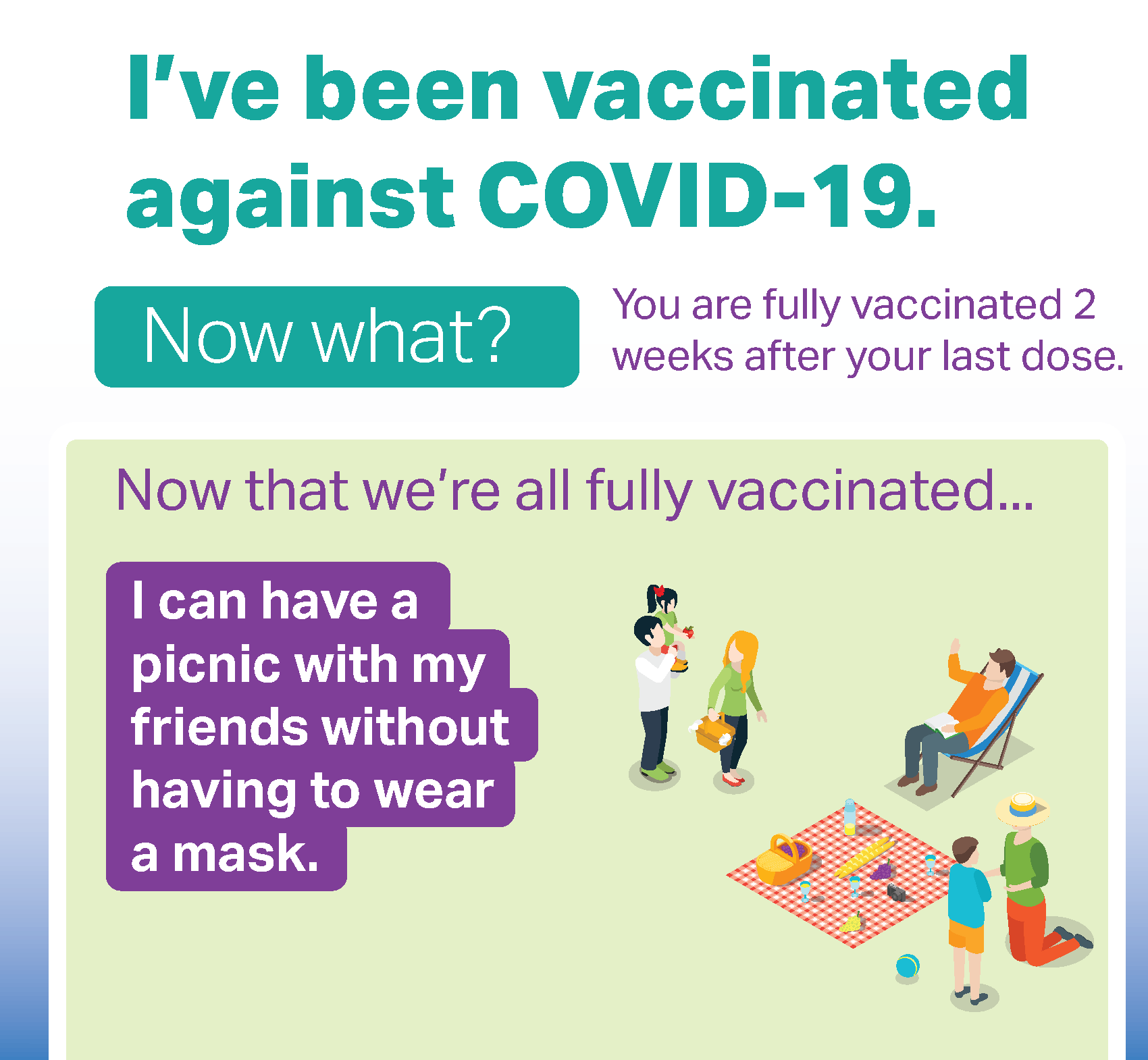


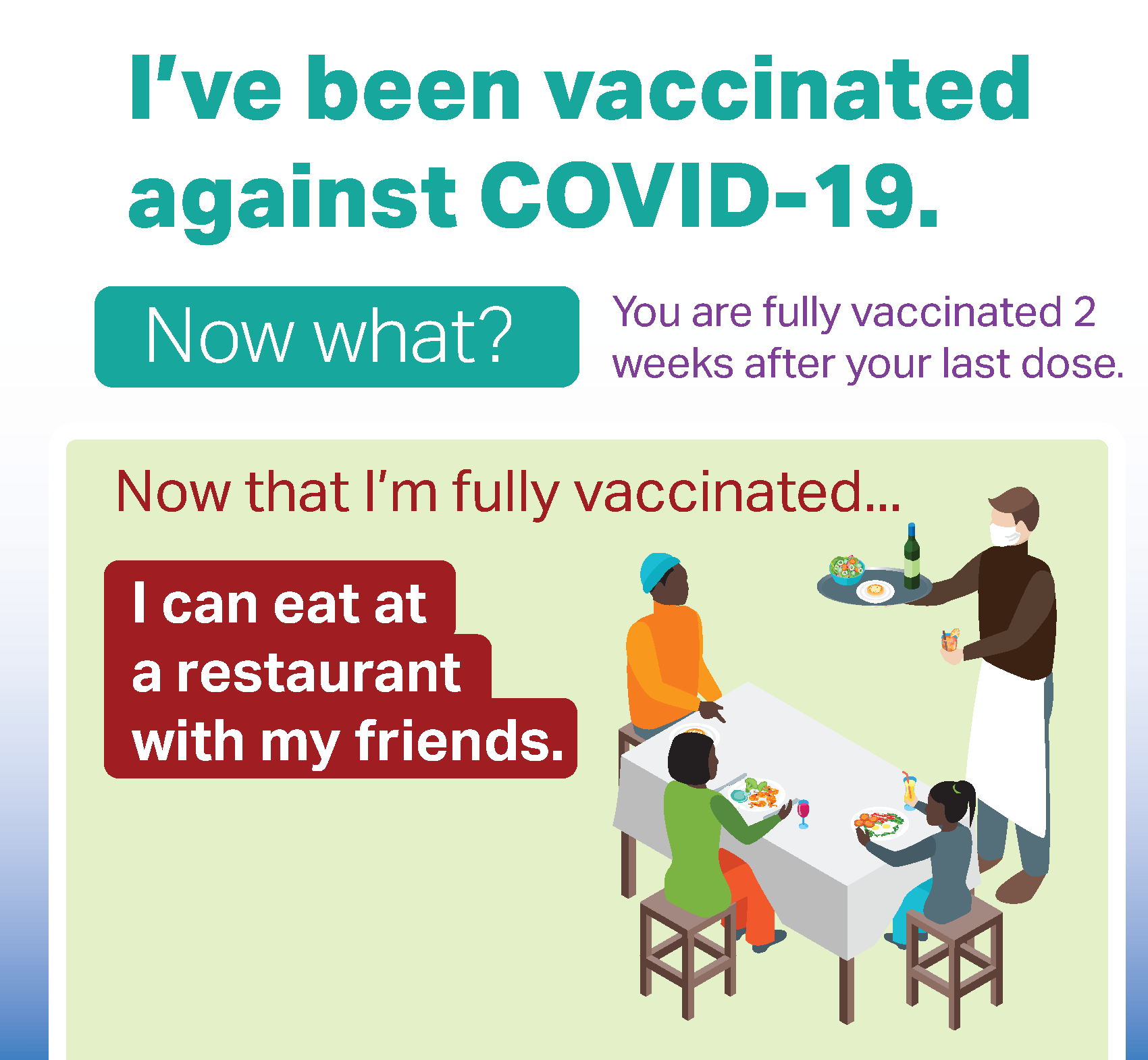


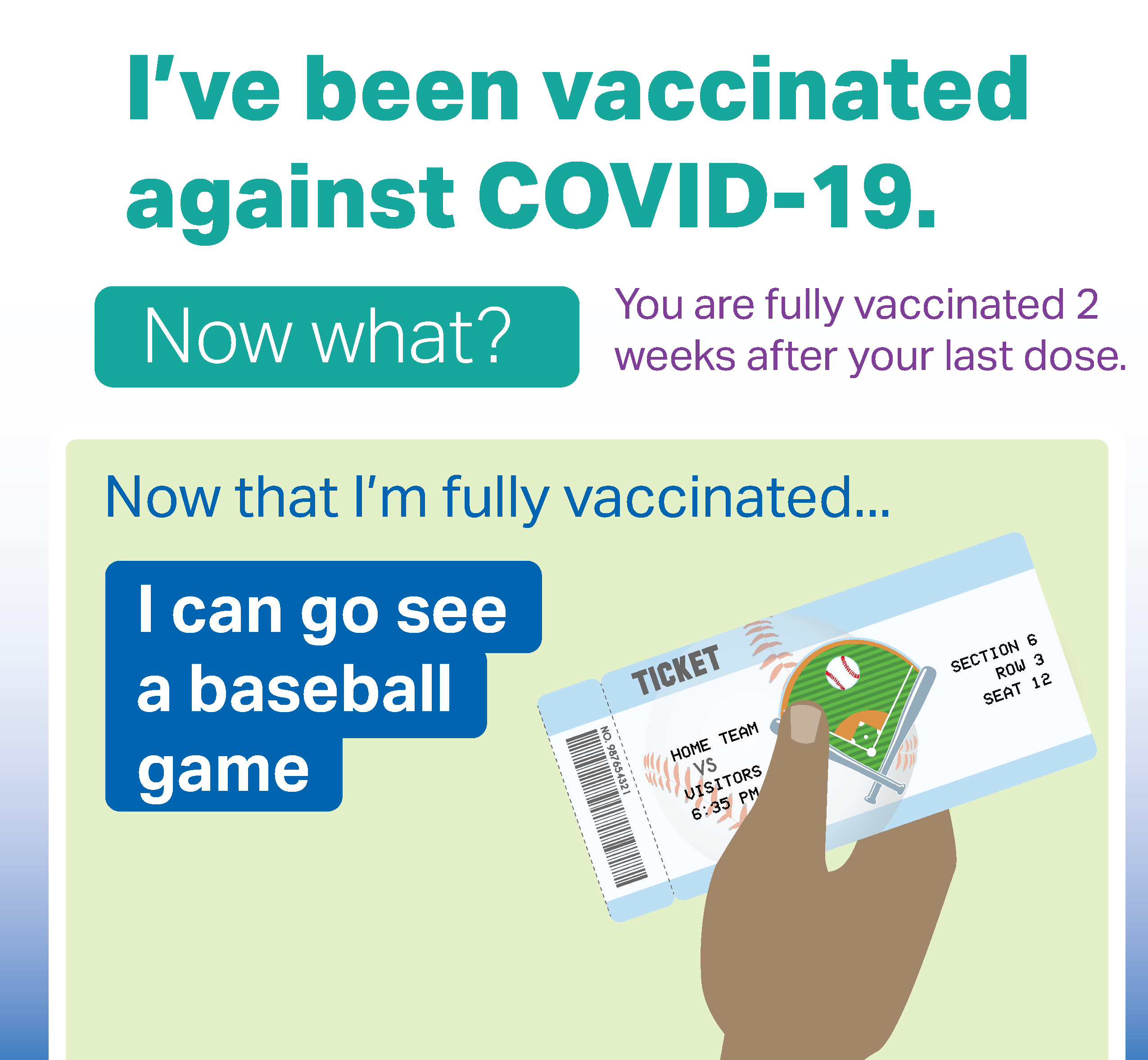


**Message Redistribution Survey**

In your last interview with the study team, you saw two messages on COVID-19 and gave your thoughts on them. To remind you of the messages, please look at them again by clicking Next Page at the bottom of each page.

[Photographs of original messages]

Next, please view six new messages on COVID-19 by clicking Next Page at the bottom of each page. After you look at them, we will ask you what you think.

[Newly created messages]

What is your overall impression of these new messages? For example, what do you think of the colors, words, or the pictures?

Do you think the creators of these new messages used your ideas from your last interview? Why or why not?
